# Supplementary material for: Providing new insights on the biphasic lifestyle of the predatory bacterium Bdellovibrio bacteriovorus through genome-scale metabolic modeling
Source: PLoS Comput Biol. 2020 Sep 14;16(9):e1007646. doi: 10.1371/journal.pcbi.1007646 (PMC7529429; doi:10.1371/journal.pcbi.1007646)
Supplement: S1 Text — (DOCX) [file pcbi.1007646.s001.docx]

**S1 Text**

**DEFINITION OF IN SILICO MEDIA**

**1.1 Definition of *in silico* rich medium**

The rich medium described below is based on the *in silico* LB medium described previously (Nogales et al., 2017) and also on conditional essential genes of *Bdellovibrio*. The default Exchange reactions were constrained as follow.

**Oxygen:** model=changeRxnBounds(model,'EX_o2(e)',-10,'l')

**Amino acids**

model=changeRxnBounds(model,'EX_val_L(e)',-5,'l')

model=changeRxnBounds(model,'EX_leu_L(e)',-5,'l')

model=changeRxnBounds(model,'EX_ile_L(e)',-5,'l')

model=changeRxnBounds(model,'EX_ser_L(e)',-5,'l')

model=changeRxnBounds(model,'EX_thr_L(e)',-5,'l')

model=changeRxnBounds(model,'EX_ala_L(e)',-5,'l')

model=changeRxnBounds(model,'EX_glu_L(e)',-5,'l')

model=changeRxnBounds(model,'EX_tyr_L(e)',-5,'l')

model=changeRxnBounds(model,'EX_gln_L(e)',-5,'l')

**Amino acids from dipeptides**

The major carbon and energy source for *Bdellovibrio* are amino acid from the breakdown of proteins, preferentially dipeptides of alanine (Odelson et al., 1982)

model=changeRxnBounds(model,'EX_ALAHIS',-5,'l')

model=changeRxnBounds(model,'EX_ALAMET',-5,'l')

model=changeRxnBounds(model,'EX_ALAASN',-5,'l')

model=changeRxnBounds(model,'EX_ALAPRO',-5,'l')

model=changeRxnBounds(model,'EX_ALAARG',-5,'l')

model=changeRxnBounds(model,'EX_ALAPHE',-5,'l')

model=changeRxnBounds(model,'EX_ALATYR',-5,'l')

model=changeRxnBounds(model,'EX_ALATRP',-5,'l')

model=changeRxnBounds(model,'EX_ALAASP',-5,'l')

model=changeRxnBounds(model,'EX_ALALYS',-5,'l')

model=changeRxnBounds(model,'EX_ALAALA',-5,'l')

model=changeRxnBounds(model,'EX_ALAGLU',-5,'l')

model=changeRxnBounds(model,'EX_ALATHR',-5,'l')

model=changeRxnBounds(model,'EX_ALACYS',-5,'l')

model=changeRxnBounds(model,'EX_ALAGLY',-5,'l')

model=changeRxnBounds(model,'EX_ALAILE',-5,'l')

model=changeRxnBounds(model,'EX_ALASER',-5,'l')

model=changeRxnBounds(model,'EX_ALAVAL',-5,'l')

model=changeRxnBounds(model,'EX_ALAGLN',-5,'l')

**Vitamins.** This medium only provides the auxotrophic metabolites of *B. bacteriovorus* HD100*.*

**Folic Acid (B9):** model=changeRxnBounds(model,'EX_thf(e)',-0.1,'l')

**Pantotenate:** model=changeRxnBounds(model,'EX_ptth(e)',-0.1,'l')

**Piridoxal phosphate:** model=changeRxnBounds(model,'EX_pdx5p(e)',-0.1,'l')

**Biotine (B8):** model=changeRxnBounds(model,'EX_btn(e)',-0.1,'l')

**Lipoate:** model=changeRxnBounds(model,'EX_lipoate(e)',-0.1,'l')

**1.2 Definition of *in silico* minimal medium**

In order to define an *in silico* minimal medium, the lower bounds of several exchange reactions were constrained as follows and only the essential reactions have been included.

**Oxygen:** model=changeRxnBounds(model,'EX_o2(e)',-10,'l')

**Essential amino acids**

model=changeRxnBounds(model,'EX_val_L(e)',-0.1,'l')

model=changeRxnBounds(model,'EX_leu_L(e)',-0.1,'l')

model=changeRxnBounds(model,'EX_ile_L(e)',-0.1,'l')

model=changeRxnBounds(model,'EX_ser_L(e)',-0.1,'l')

**Dipeptides.** The rest of amino acid will be providing in form of dipeptides

model=changeRxnBounds(model,'EX_ALAHIS',-0.1,'l')

model=changeRxnBounds(model,'EX_ALACYS',-0.1,'l')

model=changeRxnBounds(model,'EX_ALAMET',-0.1,'l')

model=changeRxnBounds(model,'EX_ALAASN',-0.1,'l')

model=changeRxnBounds(model,'EX_ALAPRO',-0.1,'l')

model=changeRxnBounds(model,'EX_ALAARG',-0.1,'l')

model=changeRxnBounds(model,'EX_ALAPHE',-0.1,'l')

model=changeRxnBounds(model,'EX_ALATYR',-0.1,'l')

model=changeRxnBounds(model,'EX_ALATRP',-0.1,'l')

Threonine and glycine will be obtained from serine. Aspartic acid and lysine will be obtained from asparagine. Tyrosine will be obtained from phenylalanine.

**Vitamins.** This medium only provides the auxotrophic metabolites of *B. bacteriovorus* HD100*.*

**Folic Acid (B9):** model=changeRxnBounds(model,'EX_thf(e)',-0.1,'l')

**Pantotenate:** model=changeRxnBounds(model,'EX_ptth(e)',-0.1,'l')

**Piridoxal phosphate:** model=changeRxnBounds(model,'EX_pdx5p(e)',-0.1,'l')

**Biotine (B8):** model=changeRxnBounds(model,'EX_btn(e)',-0.1,'l')

**Lipoate:** model=changeRxnBounds(model,'EX_lipoate(e)',-0.1,'l')

Definition of *in silico* CAV medium to simulate the growth of BdQ10

In the following, a detailed *in silico* composition of the CAV medium used to verify the possible growth of *Bdellovibrio* cells in CAV medium axenically with *i*CH457 metabolic model, is described. The default Exchange reactions were constrained as follow

**Oxigen:** model=changeRxnBounds(model,'EX_o2(e)',-10,'l')

**Amino acids**

The amount of each amino acid is select based on the experimental data obtained from the target analysis by GC-MS.

model=changeRxnBounds(model,'EX_ala_L(e)',-0.018,'l')

model=changeRxnBounds(model,'EX_val_L(e)',-0.011,'l')

model=changeRxnBounds(model,'EX_leu_L(e)',-0.011,'l')

model=changeRxnBounds(model,'EX_ile_L(e)',-0.01,'l')

model=changeRxnBounds(model,'EX_pro_L(e)',-0.004,'l')

model=changeRxnBounds(model,'EX_gly(e)',-0.01,'l')

model=changeRxnBounds(model,'EX_ser_L(e)',-0.02,'l')

model=changeRxnBounds(model,'EX_thr_L(e)',-0.009,'l')

model=changeRxnBounds(model,'EX_asp_L(e)',-0.02,'l')

model=changeRxnBounds(model,'EX_glu_L(e)',0.012,'l')

model=changeRxnBounds(model,'EX_phe_L(e)',-0.0064,'l')

model=changeRxnBounds(model,'EX_lys_L(e)',-0.0032,'l')

model=changeRxnBounds(model,'EX_arg_L(e)',-0.0028,'l')

model=changeRxnBounds(model,'EX_trp_L(e)',-0.001,'l')

model=changeRxnBounds(model,'EX_tyr_L(e)',-0.00248,'l')

**Dipeptides**

Conditional essential genes found in Barabote et al., 2009.

model=changeRxnBounds(model,'EX_ALAHIS',-0.0001,'l')

model=changeRxnBounds(model,'EX_ALACYS',-0.0001,'l')

model=changeRxnBounds(model,'EX_ALAMET',-0.0001,'l')

model=changeRxnBounds(model,'EX_ALAASN',-0.0001,'l')

model=changeRxnBounds(model,'EX_ALATYR',-0.01,'l')

model=changeRxnBounds(model,'EX_ALATRP',-0.01,'l')

**Vitamins.** This medium only provides the auxotrophic metabolites of *B. bacteriovorus* HD100*.*

**Folic Acid (B9):** model=changeRxnBounds(model,'EX_thf(e)',-0.1,'l')

**Pantotenate:** model=changeRxnBounds(model,'EX_ptth(e)',-0.1,'l')

**Piridoxal phosphate:** model=changeRxnBounds(model,'EX_pdx5p(e)',-0.1,'l')

**Biotine (B8):** model=changeRxnBounds(model,'EX_btn(e)',-0.1,'l')

**Lipoate:** model=changeRxnBounds(model,'EX_lipoate(e)',-0.1,'l')

**Other chemicals included in all the media based on Nogales et al., 2017**

| **Reaction Name** | **Reaction Description** | **Lower bond (mmol·gDW^-1^·h^-1^)** | **Lower bond (mmol·gDW^-1^·h^-1^)** |
| --- | --- | --- | --- |
| EX_co2(e) | Exchange of CO_2_ | -30 | 1000 |
| EX_fe2(e) | Exchange of Fe_2_ | -30 | 1000 |
| EX_h(e) | Exchange of H | -50 | 1000 |
| EX_h2o(e) | Exchange of H_2_O | -30 | 1000 |
| EX_hco3(e) | Exchange of HCO_3_ | -30 | 1000 |
| EX_na1(e) | Exchange Na | -30 | 1000 |
| EX_nh4(e) | Exchange NH_4_ | -30 | 1000 |
| EX_pi(e) | Exchange Pi | -30 | 1000 |
| EX_so4(e) | Exchange SO_4_ | -30 | 1000 |
