# Supplementary figures and images for: Providing new insights on the biphasic lifestyle of the predatory bacterium Bdellovibrio bacteriovorus through genome-scale metabolic modeling
Source: PLoS Comput Biol. 2020 Sep 14;16(9):e1007646. doi: 10.1371/journal.pcbi.1007646 (PMC7529429; doi:10.1371/journal.pcbi.1007646)

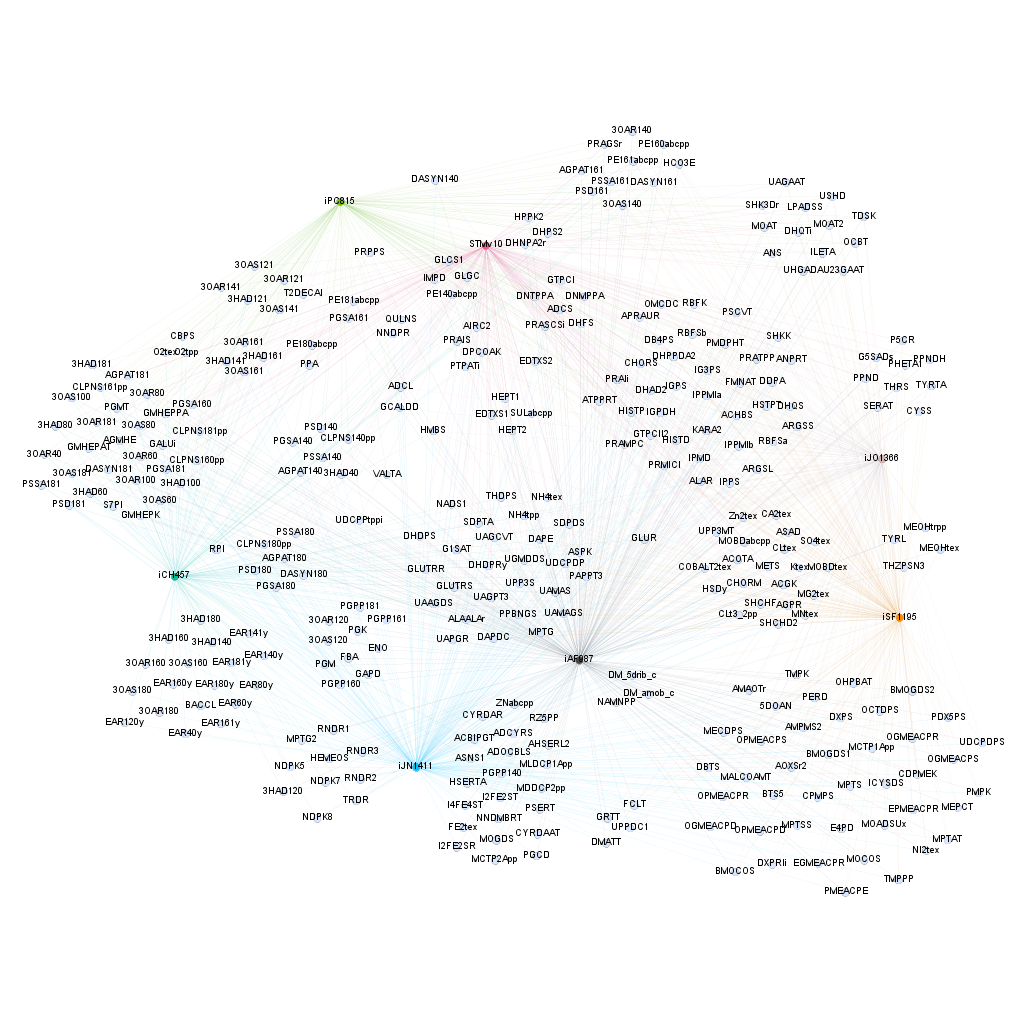

Supplement: S1 Fig — Essential reactions were grouped associated with the metabolic model. iPC815 (green), STMv10 (Pink), iJO1366 (brown), iCH457 (dark green), iAF987 (grey), iSF1195 (Orange), iJN1411 (blue). (PNG) [file pcbi.1007646.s003.png]
