# Supplementary material for: Providing new insights on the biphasic lifestyle of the predatory bacterium Bdellovibrio bacteriovorus through genome-scale metabolic modeling
Source: PLoS Comput Biol. 2020 Sep 14;16(9):e1007646. doi: 10.1371/journal.pcbi.1007646 (PMC7529429; doi:10.1371/journal.pcbi.1007646)
Supplement: Attachment — Submitted filename: Response to the referees_ Herencias et al.odt [file pcbi.1007646.s011.odt]

We really thank the reviewers for their detailed review of our manuscript. We think that their suggestions were very valuable for increase the understanding and scope of our piece. 
Below, we state point-by-point answer to all the comments of the reviewers.

Comments to the Authors:
Reviewer #1:
This manuscript by Herencias et al reports the construction of a metabolic model for the predatory bacterium Bdellovibrio bacteriovorus. Findings highlight a higher number of amino acid auxotrophies than previously known, while nucleotides and phospholipids can be synthesised de novo by Bdellovibrio.
Experimental validation of some of the model’s predictions (i.e. growth rates in various media) was done using host-independent (HI) mutant of Bdellovibrio, based on the assumption that the mutation leading to host-independency does not affect metabolism. Since the model was based on wild-type genomic and metabolic data, and because it described fairly well the metabolic activity of HI cells, it questions the obligate predatory lifestyle of Bdellovibrio from a metabolic point of view, which is interesting and intriguing. 
The authors also constructed metabolic models specific for AP and GP stages by adding in silico constraints to their initial global model (nutrient availability, biological objective, gene expression profile from published RNAseq data).

Major comments:
Query 1: Figure 4: what is the statistical significance of the growth rate differences/similarities?
Answer 1: The statistically significant differences of the two groups of data (in silico and in vivo growth rates) were calculated using two-way ANOVA followed by Bonferroni test and all the comparisons were found non-significant (P<0.05). This information has been included in the figure’s legend.

Q2: Lines 479-481: from the results section, I had understood that the investment of most resources to ATP generation was an assumption/input (qualified as “biological objective”) for the generation of the iCHAP model (see for ex. lines 366-368), not a conclusion/output from that model. Same remark for next line concerning GP. Please clarify.
A2: Condition-specific models, iCHAP and iCHGP, were constructed by constraining iCH457 using available transcriptomic data and under the assumption of maximizing ATP and growth, respectively.  However, the analysis of the allowed space in each model was performed using random MonteCarlo sampling. This approach is based on that random set of points, which are chosen from the solution space to act as surrogate for the entire space. This means that many of the properties that can be calculated for one candidate solution can then be calculated for point throughout the entire space. The obtained properties can be evaluated in statistical manner where we obtain the mean value of flux through each reaction. Therefore, this analysis allows the study of the metabolic solution space of two situations independently of the objective. See also general reply to Reviewer 2. In any case, we agree with the reviewer in that the initial biological objective assumption could bias the results and we have nuanced our statements.  In addition, a more expanded explaining of the methodology used has been included in Materials and method section.

“The development of iCH457, iCHAP and iCHGP has provided a computational framework for a better understanding of the physiological and metabolic versatility of BALOs and other predatory bacteria. These models have provided a mechanistic explaining of the required metabolic shift between the different phases. Thus, metabolic fluxes estimations during AP and GP in absence of objective using random sampling are fully compatible with the expected biological objective in these phases e.g., ATP production and growth, respectively. For instance, during GP, several metabolic pathways become inactive, allowing carbon flux distribution re-routing toward biosynthetic pathways. The TCA cycle shifts from a completely operational state during AP to an anaplerotic mode by inactivating the decarboxilative branch including citrate synthase, aconitase and isocitrate dehydrogenase.”

Q3: Figure 5: the authors could discuss what are the intracellular microorganism’s exclusive essential reactions.
A3: Table S6 provides a list of the common reactions exclusively found in the microorganism with intracellular stages in their growth cycle used in this study (Yersinia pestis CO92, Salmonella Typhimurium str. LT2 and Shigella flexneri) and B. bacteriovorus.
According to the referee suggestion a brief description and discussion have been included.

“iii) intracellular microorganisms’ exclusive essential reactions (15 reactions), related with cell envelop metabolism, glycerophospholipid metabolism, transport and nucleotide metabolism.”

“Related with the essential reactions exclusive of the intracellular microorganism (Salmonella, Shigela, Yersinia and Bdellovibrio), it can be highlighted the relevance of the lipid synthesis. These molecules participate in crucial biological processes, including signaling and organization of the membrane of the cells. For intracellular pathogens, it has been described that lipids are also crucial for the interplay with the host cell [75]. The uptake of intracellular pathogens, such as Salmonella typhimurium or Mycobacterium tuberculosis is led by a re-organization of the lipid microdomains to avoid the degradative environment of the lysosomes. Besides, in concordance with the biphasic life cycle of this intraperplasmic predator, lipid composition determined the structural and functional integrity of the extracellular forms of pathogens [76].”


Q4: related to the assumption that HI mutants’ metabolism reflects that of the wild-type Bdellovibrio: Lines 84-87: can the authors exclude the possibility that the hit mutation has a major impact on metabolism via an indirect regulatory effect?
A4: The hit locus is linked to the genetic cluster responsible for type IV pilus formation and involved in the attachment and invasion of the prey. We have now included this information in the text completing the description of HI independent strains. In any case, although unlike, we cannot rule out the possibility of an indirect metabolic impact. Subsequently we have specifically indicated that this mutation has no direct metabolic (enzymatic) impact.

“It is worth noticing that the axenic growth of these mutant strains is given by a mutation in the host interaction (hit) locus, which has been described as being involved in regulatory and/or scaffold elements, such as type IV pilus formation and also related to the attachment and invasion of the prey [11]. This argues in favor of this mutation having no direct metabolic (enzymatic) impact. In fact, the main metabolism of these HI derivatives should not have suffered changes with respect to the wild type Bdellovibrio strains”.

 Minor comments:
Q5: Title: biphasic instead of biphasic
A5: It has been corrected.

Q6: Line 44: interest in this predatory bacterium, its complex lifestyle (…)
A6: It has been corrected.
 
Q7: Line 77: septates instead of septs
A7: It has been corrected.

Q8: Lines 78-79: whether a large array of hydrolytic enzymes is used to breach the prey OM is not known.
A8: The reviewer is right pointing that is not known whether they are used to breach the prey OM. The genome of B. bacteriovorus contains a large number of hydrolytic enzymes such as, proteases, lipases and nucleases, that are used throughout the life cycle of B. bacteriovorus for prey entry, prey killing, and the uptake of complex molecules (PMID14752164.) The sentence was deleted.

“When the prey is exhausted, B. bacteriovorus grown as a filament, septates into several daughter cells, lyses the ghost-prey’s outer cell membrane and releases into the medium [6,9].”

Q9: Line 81-82: it is unclear how HI mimics the dimorphic pattern of HD cells since there is no attack phase stage. Moreover, what is meant by differentiation here is unclear.
A9: HI derivatives strains elongate and divide mimicking the life cycle of HD cells follow fission multiple instead of the classical bipartition strategy. This concept has been clarified in the text.

“These HI predators are able to grow axenically (without prey) in a rich-nutrient medium mimicking the dimorphic pattern of elongated growth, division and the development of the host-dependent (HD) cells following a fission multiple strategy [10].”

Q10: Line 94: septates
A10: It has been replaced.

Q11: Lines 108-111: this sentence is unclear, please rephrase.
A11: The sentence has been rephrased.  

“Therefore, expanding the knowledge of this predatory bacterium is essential for the full exploitation of its unique biotechnological applications. This process would require a reliable platform supporting the rational understanding of its characteristics”

Q12: Line 219: please clarify what such an energy-saving mechanism implies
A12: The biosynthesis of new biomass building blocks requires a large amount of energy. The energy-saving concept is related with the efficiency, in term of energy invested, in this anabolism. As described in D´souza et al., loss of biosynthetic pathways often is related with the presence of the target metabolites in the environment, like in the case in B. bacteriovorus. Therefore, the ability of this predator to incorporate these metabolites directly from the prey instead to synthetize them results in an energy saving process. The main text has been modified and a brief description of this concept has been incorporated.

“This phenomenon has been traditionally explained in the context of an “energy-saving” mechanism. Evolution has promoted the loss of some genetic elements in bacteria that comprise cellular fitness and are usually related with the environment, when some metabolites are sufficiently present in the bacterial growth environment. For instance, amino acid biosynthetic pathways are sometimes partially represented as described in [57,58] where the organisms are saving the energy of biosynthetic costs.”


Q13: Line 224: please clarify how the finding that Bdellovibrio has the ability to synthesise fatty acids and nucleosides de novo, and the reported uptake from the prey, demonstrates a more efficient incorporation of components from the prey.
A13: The authors agree with the unclear relation of these concepts. The rough idea was to highlight the metabolic robustness of B. bacteriovorus which is not only able to synthetize these molecules but also incorporate them from the prey. The sentence has been removed to avoid misinterpretations.

Q14: Line 249: bacteriovorus
A14: It has been corrected.

Q15: Line 268: please briefly define what FBA means (for readers who haven’t read the methods section yet).
A15: It has been defined.

“In addition, higher in silico growth rates are often found due to the intrinsic nature and limitations of the flux balance analysis (FBA).”

Q16: Line 305: after constrained, the growth rate
A16: It has been corrected.

Q17: Line 555: gene/protein
A17: It has been changed.

Q18: Figure 2: Model draft instead of mode draft?
A18: It has been modified.

Q19: Figure 2: species-specific instead of specie-specific
A19: It has been corrected.

Q20: S1 text, page 2: major instead of mayor
A20: It has been corrected.
Q21: S1 text, Table S2: abbreviations; also consider changing “thesis” to “study”.
A21: It has been changed.
Q22: Supplementary table .xls file: read me page: present instead of presents
A22: It has been corrected.
Reviewer #2:
This article describes the metabolic reconstruction of the predatory bacterium Bdellovibrio bacteriovorus. The authors test this reconstruction by predicting the bacterium’s growth rate under different growth conditions. The unique nature of this organism makes this reconstruction a useful resource for the scientific community.
While the reconstruction is useful, there isn’t anything novel in the study. There is no novel modeling approach or significant biological insights. Upon reading the abstract and title, I had presumed this study involves a novel predator-prey interaction metabolic model, which would have been very interesting. Instead this study builds a metabolic model using well-established methods and simulates various conditions in silico. The literature validation data provided isn’t extensive either and any new insights reported in the abstract are speculative.
I think the reconstruction process could be greatly improved over its current state. A lot of information about model curation and annotation have not been provided. Finally, a major goal of the reconstruction process is to identify gaps in knowledge and directions to further curate the model. These need to be emphasized in the manuscript.
There is also circular logic in their discussion and results. For their comparison of growth phase (GP) vs attack phase (AP), the authors report that they found no flux in biosynthetic pathway in the attack phase. This is just the consequence of their objective which was maximizing ATP.
Further, they report in the discussion section that they discovered “Through detailed analysis of these specific-condition models it has been possible to conclude that during AP, B.bacteriovorus invests most of its resources in ATP generation… In contrast, GP was characterized by the biosynthesis of biomass building blocks.” But while performing FBA, they assumed that the AP phase maximizes ATP and the GP maximizes biomass! So it doesn’t make sense to conclude this result based on their assumption. If they had found this using just the transcriptomics data alone then that would have been interesting.

We thanks to the reviewer for her/his comments and suggestions.
 
We would like first to clarify the AG and GP circular logic suggested by the reviewer as we consider that it’s not a completely circular discussion.  The assumed biological objectives were used for the construction of the condition-specific models but not during models analyses since random sampling, instead of FBA was used, please see Q2 Reviewer 1. In addition, for the construction of such condition-specific models we used transcriptomic data and constrained the models based on the potential nutritional conditions likely present in each phase. Overall, our analysis showed that predicted fluxes, in the absence of objective optimization, were fully consistent with these three constraints. For this reason, we strongly disagree with the reviewer when he/she think that no novel biological insights are provided in our study. We show the dynamic metabolism required to successful survival in the extremely different environments that face Bdellovibrio. In addition, and due the nature of this predator our approach supposes the only alternative available to know, although roughly, the metabolic fluxes driven GP in this predator. Finally, we agree with the reviewer in that no novel modeling methodology is developed, but this is not the aim of this study but to provide the Bdellovibrio’s community of a high-quality metabolic model using well-established methods and to provide the first flux metabolic study of a bacterial predator.

Regarding the inclusion of additional details about model reconstruction, we have included now new information in the methods section. In addition, we have performed a detailed quality analysis using MEMOTE that guarantee the high quality of iCH457, see below.

Q23: How was reaction directionality determined? was it using literature evidence or other evidence such as basic thermodynamic estimates? This is not discussed in the manuscript
A23: Since experimental information about reaction directionality for all the reactions in the model is not available, this information was assigned based on i) literature legacy, when available and/or ii) metabolic and enzymatic databases such as Brenda, Kegg, Uniprot, and/or iii) GEMs databases such as Bigg and MetaNetX and/or iv) similar reactions included in well curated GEMs e.g., E. coli and P. putida models. This information has been now briefly detailed in the Methods section.

Q24: Does the model include growth and non-growth-associated ATP maintenance parameters? if so how were they estimated?
A24: The model includes both. Growth-associated maintenance (GAM) was included in the biomass objective function, and non-growth-associated maintenance (NGAM) as an ATP hydrolysis reaction (ATPM), see MEMOTE report (SI2). Since Bdellovibrio-specific information for GAM and NGAM is not available and taking into account the real challenge that represent its estimation in this bacterium due to its predatory life style, we taken GAM and NGAM values from well-curated model of P. putida. Although this is not an ideal scenario, this is a well-accepted option for those microorganisms without experimental information available (Thiele and Palsson, 2010).  

Q25: How were Genes-Protein-Reaction annotations done? Was protein complex information taken into account during the reconstruction process to assign GPRs?
A25: When available, GPR was assigned taking into account literature legacy. For those genes without experimental information, we assigned the GPR association based on sequence homology with genes of known function in other organisms. GPR relationships follow a Boolean logic to describe the nonlinear associations, where “and” correspond to protein complex and “or” is related with isoenzymes.
For instance, the succinyl-CoA synthase complex is represented:
SUCOAS
succinyl CoA synthetase
coa[c] + atp[c] + succ[c]  <=> adp[c] + pi[c] + succoa[c]
(Bd3810 and Bd3811)

Malic enzyme reaction is encoded by two different genes in Bdellovibrio, thus this reaction is represented:
ME2
malic enzyme NADP
nadp[c] + mal_L[c]  -> co2[c] + pyr[c] + nadph[c]
(Bd1833 or Bd1834)

This information has been now briefly detailed in the Methods section.

Q26: Regarding compartments in the model, was some sequence analysis done to determine subcellular location of different enzymes (i.e cytosol or periplasm)?
A26: No specific computational analysis was curried for determine subcellular localization, instead, compartments were assigned based on the subcellular location of homologous proteins in high-quality models (E. coli, P. putida, etc). In a few cases, available experimental information was taking into account. For instance, the glyceraldehyde 3 phosphate dehydrogenase was studied specifically in B. bacteriovorus as well as other glycolytic enzymes as described in Hespell et al.,1976 (PMID: 135759).
We have briefly detailed this point in the Methods section.

Q27: How many dead end reactions are there in the model? These could be highlighted/discussed for future model curation and to identify less studied parts of the network.
A27: The network contains a total of 97 (≈10%) dead-end metabolites including the 20 tRNA amino acids, often included in GEMs but not effectively connected (Thiele and Palsson, 2010).  We present now these dead metabolites in the Memote’s report (SI2). Many of these dead-end metabolites are related with lipids and peptidoglycan metabolisms. Also there are 408 blocked reactions in the model according MEMOTE. Again many of these unused reactions are related with lipids and peptidoglycan metabolism. Overall, it seems that there is still an incomplete modeling of cell envelope biosynthesis in the model, likely due to incomplete knowledge of this aspects in Bdellovibrio.
We have highlighted this fact in the manuscript.  

“This high number of hydrolytic reactions present in iCH457 is consistent with the important role of these enzymes in the degradation of the prey’s cell wall to penetrate into the periplasm, completion of the growth cycle and also in recycling the envelope components [49]. However, among the blocked reactions present in the model, those related with lipids and peptidoglycan metabolisms are over represented, suggesting a still incomplete cell envelope biosynthesis in the model. Therefore, this subsystem supposes a clear target for model expansion in future studies.”

Q28: the confidence scores for reactions are not provided in the spreadsheet supplement. The confidence scores are valuable for model curation. The transcriptomic and genomic evidence mentioned earlier can be used to assign these confidence scores (along with known biochemical literature).

A28: The confidence scores were available in Supplementary Table 1. We reviewed the data and incorporated the overall CS into the main text as well.

“Thus, the model of B. bacteriovorus HD100, iCH457, represents a new model within this group, which, as depicted in Table 1, provides a complete reconstruction of this important bacterial group in terms of the metabolites and reactions included (Overall confidence score = 2.1).”

Q29: How were the reactions mass- and charge balanced?
A29: Following well-established procedures (Thiele and Palsson), we balanced all the reactions in the network taking into account the charged formula of each metabolite at intracellular pH of 7.2.
As indicated in the MEMOTE report, the 99,9% of the reactions in the model are correctly mass and charge balanced. In fact, only one single reaction remains unbalanced due incomplete information of the pathway in which is involved (KAS16_BD, beta-ketoacyl_ACP synthase).
 
Q30: What other quality controls were performed during the reconstruction process? Did they check for ATP, nadh, nadph production leaks (i.e they should not be produced when there are no inputs)? thiele and palsson, 2010 have a good protocol for testing reconstructions. This can be used as a guideline for reconstructing metabolic networks.

A30: We thanks the reviewer his suggestion, in fact we have profusely used the protocol of Thiele and Palsson during the reconstruction process. Regarding quality controls, we addressed all the analysis suggested by the reviewer during the reconstruction in order to avoid loops and cycles impacting the correct performance of the model. In addition, we have now included additional quality controls by using MEMOTE quality testing platform (Nat Biotechnol. 2020 Mar;38(3):272-276. doi: 10.1038/s41587-020-0446-y.). MEMOTE, represents a unified community-based approach to ensure the formally correct definition of SBML3FBC and provides quality control and continuous quality assurance of metabolic models with tools and best practices already used in software development. As available through the MEMOTE Report (SI2), the model has passed the test by far, reaching a very high score (88.28%). In fact, iCH457 achieved up to 98.71% in the important category of model consistence. Overall, MEMOTE analysis guarantee the high quality of Bdellovibrio model. We have now highlighted this extra quality control in the text as follows:

“In order to ensure the quality of the predator model, we used the MEMOTE tool (https://memote.io/) [55] to evaluate the completeness and consistency of the metabolic network. The model's overall score was 88 %, which suggests a good model completeness (SI2). The consistency of the model scored 99%, which represent the accuracy in stoichiometry, mass and charge balances, connectivity of metabolites and reaction cycles. This analysis confirms iCH457 as a complete and detailed model. The model not scoring even higher mainly due to an incomplete annotation to outside references for all genes, metabolites and/or reactions in the GEM.  Although this can be related with some difficult to use the model with certain bioinformatics tools of script, the accuracy and usability should not be affected”.

Q31: The authors mention that there are gaps in the model. How many gaps were there and how many were filled manually and using automated tools?
A31: During the reconstruction process, a considerable number of gaps were identified in the network and revised by manual curation. Some of them were solved based on experimental evidences, and although a specific gene could not be assigned, we included the missed reactions as orphan reactions (reaction not gene-associated). Example of this includes: the enoyl ACP reductase involved in the fatty acid biosynthesis as it’s evident that lipids are synthetized in Bdellovibrio despite the gene encoding this activity is unknown.  However, many other gaps, mainly involved in amino acid biosynthesis, could not be solved, concluding that these gaps are indeed responsible of known and unknown autotrophies.
This information has been now included in the text:

“During the reconstruction process, we identified several incomplete biosynthetic pathways (lipids, amino acids, cofactors, and vitamins). Many of these metabolic gaps could be solved based on experimental evidences, and although a specific gene could not be assigned, we fill the gaps by using orphan reactions (reaction not gene-associated). Example of this includes: the enoyl ACP reductase involved in the fatty acid biosynthesis as it’s evident that lipids are synthetized in Bdellovibrio despite the gene encoding this activity is unknown.  However, many other gaps, mainly involved in amino acid biosynthesis, could not be solved, concluding that these gaps indeed could be responsible of known and unknown autotrophies, which is that are consistent with the numerous autotrophies previously reported for strain HD100 [6].”

Q32: The authors report that this reconstruction has high number of transporters and cell wall metabolism enzymes. For example, they state “In fact, this category was found to be one of the most representative in terms of number of reactions (161)”. Are these statistics higher than those observed in ecoli or other bacteria? the actual number (161) alone doesnt really convey if transporters are over-represented. This needs to be described in comparison with other organisms.
A32: iCH457 model contains 181 transport reactions that represent the 18 % of the total reactions in the model, which suppose a significant higher number of transport when compared with other δ-proteobacteria. The number of transport reactions was added in Table 1 in order to easily compare the weight of this parameter in the models related models. This comparison showed that iCHA457 has a lower amount of transport reactions than metabolically versatile bacteria such as E. coli and P. putida however, this subsystem is overrepresented when compared with other δ-proteobacteria models such as Geobacter sps and Desulfovivrio vulgaris. We have pointed out this fact in the text.

“In fact, this category was found to be one of the most representative in terms of number of reactions (181), highlighting their importance in cellular interchange compared with the transport reactions in other δ-proteobacteria metabolic models, (Table 1).”

Table 1. Comparison of the metabolic properties of iCH457 compared with other δ-proteobacteria (Geobacter spp.and Desulfovibrio vulgaris) metabolic models and with the well-establish metabolic reconstruction of P. putida (iJN1411) and E. coli (iJO1366).


Q34: The authors predicted growth and biomass yield in 5 conditions. They report 100% accuracy in predicting growth in 3 conditions and 70% accuracy in 2 conditions. I think the term accuracy is confusing and misleading in this case. Instead they should say ‘the model predicted 70% of the actual value’. Please include a pvalue for the rank correlation reported as it is a function of the sample size.

A34: The text was modified to remove the term accuracy. Statistics of these calculations were included in the text (See also Q1).

Q35: table s2 in the supplementary text: What’s the literature evidence for these reannotations? this should be included in the table. No references or evidence code is provided in s1 table for the curation of these reactions

Q35: In order to simplify the data management, we have moved the table containing the reannotation information from supplementary text to the excel file containing the Supplementary tables, Table S2. As mentioned above, many metabolic gaps were found in fatty acid and cell envelope during the reconstruction process, and not all of them could be solved Q27. In order to complete the modeling of these subsystems, a detailed manual curation process was performed as the initial annotation of many genes potentially involved in these processes was very unspecific and/or vague. The reannotation effort therefore should be understood in this context (See also Q31). We re-assign and/or update the function of up to 10 genes by sequence homology using Blast-n and Blast-p and/or by network contextualization and/or genome context. For instance, the gene Bd1852 was initially annoted as an unspecific, Enoyl-CoA hydratase in the genome of Bdellovibrio. Homology-based draft reconstruction identified this gene as encoding for one of the 3 methylglutaconyl CoA hydratases acting in the branched chain amino acids. Finally, manual gap-filling analysis suggested the participation of this gene as a putative Methylmalonyl-CoA decarboxylase as well. This hypothesis could be validated in this case based on literature legacy (Nguyen et al., 2008). The role of Bd1836 also could be validated including literature evidences (Lambert et al., 2010). These two references have been now included in the table and we have modified the text for better understanding as follows.

“Moreover, during the manual curation process, we confirmed (by sequence homology and further metabolic contextualization the function of several genes related to amino acids metabolism and the hydrolytic enzymes involved (Tables S1 and S2). For instance, gene bd0950, annotated as an unspecific acetyltransferase, was specifically associated with an UDP 2,3-diamino-2,3-dideoxy-D-glucose acyltransferase, while gene bd2095, firstly annotated as encoding for an acetyl-CoA C-acetyltransferase, was unequivocally re-annotated as a 3-ketoacyl-CoA thiolase. Similarly, bd1852 which was initially annoted as an unspecific Enoyl-CoA hydratase in the genome of Bdellovibrio, the homology-based draft reconstruction identified this gene as encoding for one of the 3 methylglutaconyl CoA hydratases acting in the branched chain amino acids metabolism. Finally, manual gap-filling analysis and literature legacy suggested the participation of this gene as a putative Methylmalonyl-CoA decarboxylase [47]."

Other comments
Q36: There’s a typo in the title. Biphasic is misspelled.
A36: It has been corrected.

Q37: The abstract uses unnecessary adverbs like ‘unprecedented’, ‘solid’.
A37: These adverbs have been removed.

Q38: Figure 2 would be more informative if it had statistics rather than just words and cartoons describing the process.
A38: Figure 2 has been modified to provide more information of the workflow.

Q39: Notes in s1 table in the spreadsheet are in Spanish
A39: The notes have been removed from the final version of the manuscript, since they are notes during the reconstruction process.
